# Clinical outcomes of different autografts used for all-epiphyseal, partial epiphyseal or transphyseal anterior cruciate ligament reconstruction in skeletally immature patients – a systematic review

**DOI:** 10.1186/s12891-023-06749-4

**Published:** 2023-08-03

**Authors:** S Verhagen, M Dietvorst, EJLG Delvaux, MC van der Steen, RPA Janssen

**Affiliations:** 1 Department of Orthopaedic Surgery & Trauma, PO box, Máxima, Eindhoven, 5600 PD MC The Netherlands; 2 MMC Academy, Máxima, Veldhoven, MC The Netherlands; 3 Department of Orthopaedic Surgery & Trauma, Catharina Hospital Eindhoven, PO box 1350, Eindhoven, 5602 ZA The Netherlands; 4 Orthopaedic Biomechanics, Department of Biomedical Engineering, Eindhoven University of Technology, Eindhoven, The Netherlands; 5 Chair Value‑Based Health Care, Department of Paramedical Sciences, Fontys University of Applied Sciences, Eindhoven, The Netherlands

**Keywords:** Anterior cruciate ligament, Reconstruction, Graft, Hamstring, Quadriceps, Patella, BPTB, Skeletally immature, Children, Open physes

## Abstract

**Background:**

Different types of grafts can be used for anterior cruciate ligament reconstruction (ACLR). There is little published data regarding skeletally immature patients. The purpose of this systematic review was to assess the clinical outcomes and complications for different autograft types used in all-epiphyseal, transphyseal and partial epiphyseal/hybrid ACLR in skeletally immature children and adolescents.

**Methods:**

PubMed, Embase and Cochrane databases were systematically searched for literature regarding ACLR using hamstrings, quadriceps or bone-patellar-tendon-bone (BPTB) autografts in skeletally immature patients. Studies were included if they examined at least one of the following outcomes: graft failure, return to sport(s), growth disturbance, arthrofibrosis or patient reported outcomes and had a minimum follow-up of 1 year. Case reports, conference abstracts and studies examining allografts and extra-articular or over-the-top ACL reconstruction techniques were excluded. Graft failure rates were pooled for each graft type using the quality effects model of MetaXL. A qualitative synthesis of secondary outcomes was performed.

**Results:**

The database search identified 242 studies. In total 31 studies were included in this review, comprising of 1358 patients. Most patients (81%) were treated using hamstring autograft. The most common used surgical technique was transphyseal. The weighted, pooled failure rate for each graft type was 12% for hamstring tendon autografts, 8% for quadriceps tendon autografts and 6% for BPTB autografts. Confidence intervals were overlapping. The variability in time to graft failure was high. The qualitative analysis of the secondary outcomes showed similar results with good clinical outcomes and low complication rates across all graft types.

**Conclusions:**

Based on this review it is not possible to determine a superior graft type for ACLR in skeletally immature. Of the included studies, the most common graft type used was the hamstring tendon. Overall, graft failure rates are low, and most studies show good clinical outcomes with high return to sports rates.

**Supplementary Information:**

The online version contains supplementary material available at 10.1186/s12891-023-06749-4.

## Background

The incidence of anterior cruciate ligament (ACL) ruptures in children is increasing, as well as the number of ACL reconstructions (ACLR) and graft failures in this age group [11, 19–21, 67, 69]. Different types of grafts can be used for ACL reconstruction, which can be divided in autograft and allograft. Graft choice for ACL reconstruction in skeletally immature patients are influenced by the surgeon’s preference, surgical technique, patient size and the remaining skeletal growth [11, 12, 49, 65].

The most commonly used autograft types are hamstring, quadriceps and patellar tendon grafts [5, 70]. Previous research comparing the different graft types in adults or adolescents with closed physes showed contrasting results [52, 54, 56, 64]. Some studies showed a lower graft failure rate in bone-patellar tendon-bone autografts (BPTB) compared to hamstring autografts [52, 54]. Contrasting, a cohort study by Snaebjörnsson et al. (2019) including 18,425 patients from the Swedish and Norwegian National Knee Ligament Registries showed no significant different revision rate due to graft failure between hamstring and patellar tendon autografts [64].

There is little published data on clinical outcomes of different graft types in children and adolescents with open physes [17]. Common techniques used for ACL reconstruction in children with open physes are transphyseal, all-epiphyseal and partial epiphyseal (hybrid) [3]. Another common technique is the over-the-top procedure, which is a combined intra- and extra-articular reconstruction with an iliotibial band autograft or hamstring tendon autograft [28, 69]. It has been previously observed that the use of autograft instead of allograft decreases the rate of graft failure in pediatric patients [11]. Given this outcome, the use of allograft has widely been discouraged and will not be investigated in this review. Whilst several studies describe clinical outcomes after pediatric ACL reconstruction, the available literature has not yet been systematically reviewed to determine which specific autograft type provides the best results regarding stability, return to sports (RTS) and complications such as graft failure, arthrofibrosis and growth disturbances, in skeletally immature children and adolescents undergoing transphyseal, all-epiphyseal or partial epiphyseal ACL reconstruction.

The aim of this systematic review is to assess the clinical outcomes and complications for different autograft types used in all-epiphyseal, transphyseal and partial epiphyseal/hybrid anterior cruciate ligament reconstructions in skeletally immature children and adolescents, in order to provide the surgeon with sufficient information when choosing a graft type and to stimulate personalized medicine. In line with findings in the adult population [27, 53, 54], we expect that the hamstrings tendon autograft will be most frequently used for ACLR in children and adolescents. Furthermore we hypothesize that the quadriceps tendon and BPTB autograft do not show inferior clinical outcomes as compared to hamstrings tendon autograft.

## Methods

This systematic review was performed according to the Preferred Reporting Items for Systematic Reviews and Meta-Analyses (PRISMA) guidelines [60] and registered with Prospective register of systematic reviews (PROSPERO) (CRD42022307073).

### Literature search

Pubmed, Embase and Cochrane were searched from inception to December 20, 2021. The specific search strategy was created by a consultant with expertise in systematic review searching. A literature search using medical subject headings (MeSH) and text words related to ACL graft reconstruction in children/adolescents with open physes was used. Key search terms included skeletally immature children/adolescents, open growth plates, open physes, anterior cruciate ligament reconstruction, transphyseal, all-epiphyseal, partial epiphyseal/hybrid, physeal sparing technique, quadriceps, patellar tendon, bone patellar bone, graft type, autograft, hamstring, re-ruptures, graft failure, return to sport(s), growth disturbances, arthrofibrosis. The full search is described in Appendix 1. After the study selection, the reference lists of included studies were scanned to ensure literature saturation.

### Study selection

All studies were screened by title and abstract and in a second phase using full text by two independent reviewers using the Rayyan software program [47]. Disagreements were settled by a third reviewer. Only original research studies related to ACL reconstructions (all-epiphyseal, partial epiphyseal or transphyseal technique) in skeletally immature children and adolescents (with open physes) with use of autografts (for example hamstring, quadriceps or BPTB) were included if they studied at least one of the following outcomes: graft failure, return to sport(s), growth disturbance, arthrofibrosis or patient reported outcomes and had a minimum follow-up of 1 year. Studies addressing both data of patients with open and closed physes were included if data on the result of treatment were reported separately for each patient group. If results were not reported separately, the authors were contacted and requested to provide the separate data. Only studies written in the English or Dutch language were included. Case reports, conference abstracts and studies examining allografts were excluded. Studies examining extra-articular tenodesis or over-the-top ACL reconstruction techniques were excluded due to the extra-articular nature of the procedure, which differs from the purely intra-articular ACLR techniques and can therefore not be rightfully compared to each other.

### Data extraction

Data extraction was performed by two independent reviewers and recorded into a spreadsheet. After comparison and discussion of both spreadsheets, a database containing the final extracted data was formed. Study characteristics recorded included authors, publication year, design, inclusion and exclusion criteria, bone age analysis or tanner staging, outcome measurements, number of study subjects, graft type, ACLR technique and rehabilitation program. Specific patient characteristics concerning mean age of study subjects, sex, body mass index (BMI) and timing of surgery were also recorded.

### Risk of bias assessment

Risk of bias was assessed with the Methodological Index for Non-Randomized Studies (MINORS) score [62]. Each methodological item was scored as 0 (not reported), 1 (reported but inadequate) or 2 (reported and adequate). Non-comparative studies could earn a maximum score of 16 and comparative studies of 24, respectively, with a higher score indicating a higher study quality and lower risk of bias.

### Outcomes

The primary outcome was graft failure, determined by Magnetic Resonance Imaging (MRI), arthroscopy and/or clinical diagnosis. Secondary outcomes were clinical outcomes and growth disturbances. The following clinical outcome measurements were included in the review: Knee stability, arthrofibrosis, growth disturbances, patient reported outcome measures (PROMs) and return to sports (RTS). The definition of arthrofibrosis varied per study and was usually measured through physical examination [46]. Previous studies have reported RTS as a percentage of how many patients returned to their prior level of sports activity. Commonly used PROMs for subjective functional knee outcomes were the Lysholm score or (pedi-) International Knee Documentation Committee (IKDC) [10, 57]. All types of measurements were taken into account. Only growth disturbances that are measured radiologically in mm or cm (shortening or overgrowth) or degrees (valgus, varus or recurvatum) were evaluated.

### Statistical analysis

Graft failure rates (primary outcome) were pooled for each graft type using the quality effects model of MetaXL [18]. This model gives lower weights to studies of lower quality. The first 8 items -applicable to all included studies- of the MINORS score were used to determine the study quality. Only studies presenting similar definitions of re-rupture or graft failure were included in this pooling. In addition, a qualitative synthesis of secondary outcomes was performed. Secondary outcomes evaluated in a similar manner regarding definition or method were included in this synthesis. Outcomes across four or more studies were reported as ranges, and outcomes assessed in three or fewer studies were reported individually.

## Results

**Fig. 1:**
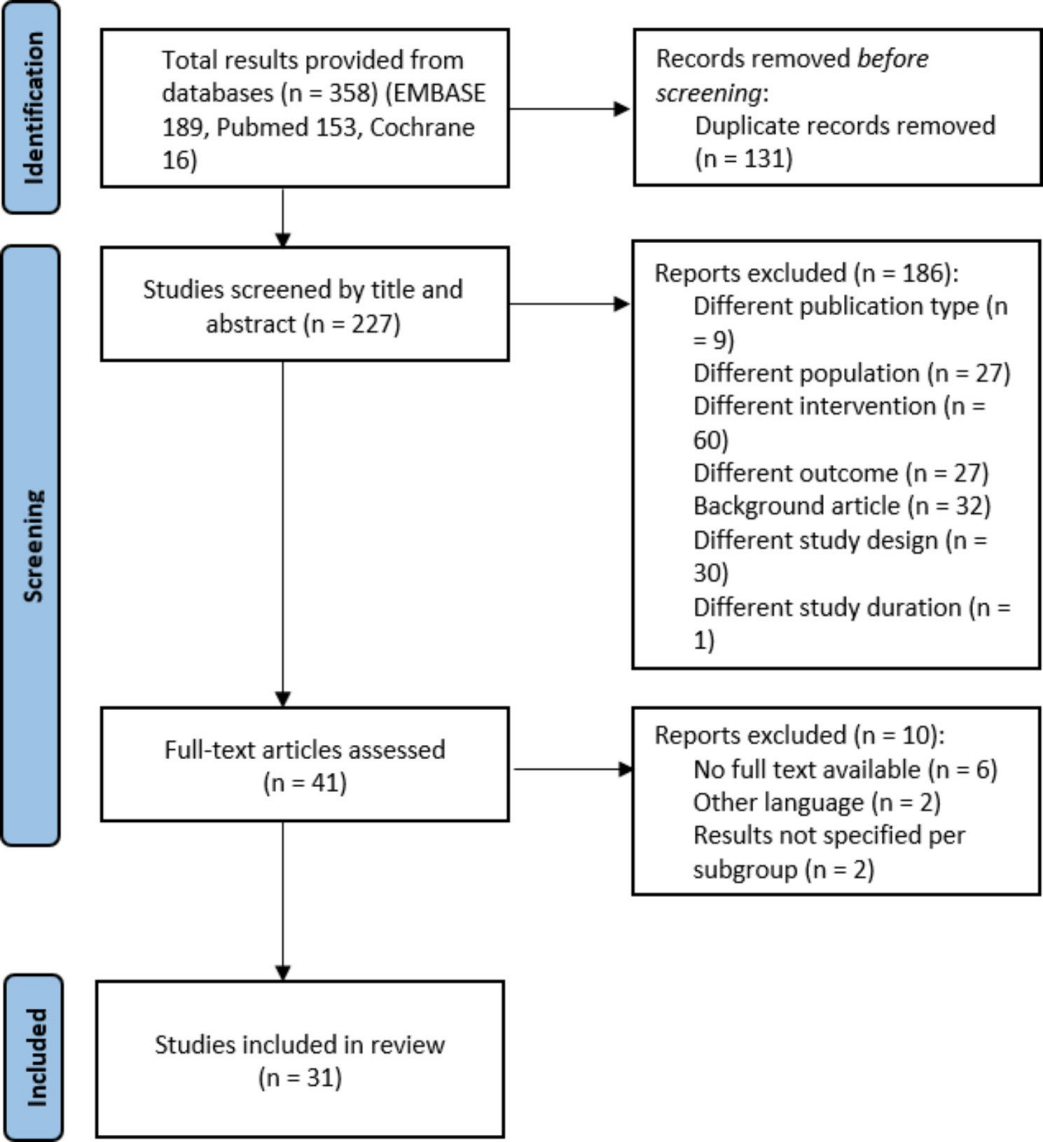
Flowchart which shows the study screening and inclusion process

The database search identified 242 studies. After the removal of duplicates, screening of abstracts and full texts, 31 studies were included in this review (Fig. 1). These studies evaluated in total 1358 patients. The average age of the participants ranged from 11 to 15 years and the individual study populations consisted in 0–61% of female patients. The average duration of follow-up ranged from 21 months to 10 years (Table 1).

**Table 1:** Study characteristics and study bias scores of the included studies

Study	Design	Graft type(s)	ACLR technique	Number of patients	Mean age in years	Sex (female%)	Mean bone age in years	Tanner stage	Mean follow-up period	Total MINORS score
Anderson [2]	Case series	Hamstring	All epiphyseal	12	13.3	17	.	I 25%, II 33%, III 42%	4.1 years	8
Astur [4]	Case control	Hamstring	Transphyseal	52	Intact ACL 13.6, graft failure ACL 14.4	Intact ACL 53%, ACL graft failure 28%	.	Intact ACL II 26%, III 32%, IV 42%. ACL graft failure, II 17%, III 44%, IV 39%	24 months	17
Calvo [6]	Case series	Hamstring	Transphyseal	27	13	41	.	II 11%, III 22%, IV 67%	10 years	10
Chambers [7]	Retrospective case series	Hamstring	Partial transphyseal	24	12.3	21	.	.	31.5 months	10
Cohen [8]	Retrospective case series	Hamstring	Transphyseal	26	13.3	58	.	I or II 19%, III 35%, IV 46%	45 months	8
Cordasco [10]	Case series	Hamstring	All-epiphyseal	23	12.2	26	12.2	.	32.1 months	12
Faunø[22]	Prospective cohort	Hamstring	Transphyseal	33	11.7	.	.	.	68 months	8
Gebhard [24]	Retrospective cohort	Hamstring, quadriceps	Transphyseal	28 (HS 16, QT 12)	11.9	48	.	Immature I, II & III	32 months	10
Gicquel [26]	Prospective cohort	Hamstring, quadriceps	.	71	13.4	.	.	.	2 years	12
Graziano [29]	Prospective case series	Hamstring	All epiphyseal (60%), Partial transphyseal (40%)	42	12	29	.	.	.	6
Guzzanti [31]	Prospective case series	Hamstring	Partial transphyseal	10	13.6	0	13.5	II 30%, III 70%	40 months	9
Koch [33]	Retrospective case series	Hamstring	All epiphyseal	12	12.1	17	.	.	54 months	11
Kocher [34]	Retrospective case series	Hamstring	Transphyseal	61	14.7	61	14.4	III 97%, IV 3%	3.6 years	9
Kohl [35]	Prospective case series	Quadriceps	Transphyseal	15	12.8	20	.	II 40%, III 47%, IV 13%	4.1 years	9
Kopf [36]	Prospective case series	Hamstring	Transphyseal	14	14.4	43	.	II and III 100%	7 years	9
Lemaitre [37]	Retrospective case series	Hamstring	Transphyseal	13	13.7	.	13.6	.	15 months	8
Mauch [39]	Retrospective cohort	Quadriceps	Transphyseal	49	13	43	.	.	5 years	10
McCarroll [40]	Prospective case series	BPTB	Transphyseal	60	14.2	52	.	III & IV (100)	4.2 years	10
McIntosh [41]	Retrospective case series	Hamstring	Transphyseal	16	13.6	31	.	.	41.1 months	10
Memeo [42]	Case series	BPTB	Transphyseal	10	14.4	20	.	III (100)	24.9 months	6
Nelson [44]	Retrospective registry study	Hamstring, BPTB	.	443 (HS 388, BPTB 55)	14.9	36	.	.	2.9 years	10
Nikolaou [45]	Retrospective case series	Hamstring	Transphyseal	94	13.7	40	.	I 26%, II 44%, III 27%, IV 3%	38 months	10
Pennock [48]	Retrospective cohort	Hamstring, quadriceps	Transphyseal	83 (HS 56, QT 27)	14.8 (HS 14.8 QT 14.8)	HS 31%, QT 32%	14.6 (HS 14.6 QT 14.4)	.	HS 2.8 years, QT 2.4 years	16
Razi [50]	Prospective cohort	Hamstring	Partial transphyseal or transphyseal	18	15	28	.	II-III 41.6%, IV 42.1%	24 months	16
Redler [51]	Retrospective case series	Hamstring	Transphyseal	18	14.2	33	14	.	43,4 months	9
Sasaki [55]	Retrospective cohort	Hamstring	All-epiphyseal	18	12.4	56	.	.	42 months	20
Seon [59]	Case series	Hamstring	Transphyseal	11	14.7	0	.	II 18%, III 73%, IV 9%	77.7 months	8
Shelbourne [61]	Retrospective case series	BPTB	Partial transphyseal	16	14.8	31	.	III 44%, IV 56%	3.4 years	11
Smoak [63]	Retrospective case series	Hamstring	Transphyseal	9	12.9	22	.	.	4.6 years	8
Wall [66]	Retrospective case series	Hamstring	All epiphyseal	27	11.4	15	11.8	.	3.8 years	9
Willson [68]	Retrospective case series	Hamstring	Partial transphyseal	23	13	26	13.6	.	21 months	10

ACL; anterior cruciate ligament, ACLR; anterior cruciate ligament reconstruction, BPTB: bone-patellar-tendon-bone, HS: hamstring, BPTB: bone-patellar-tendon-bone, QT: quadriceps, MINORS score; The Methodological Index for Non-Randomized Studies, if a ‘’.’’ is used, the study did not report this characteristic

The majority of the included studies (n = 26) investigated ACLR using hamstring tendon autograft (n = 1042 patients, 81%). BPTB and quadriceps tendon autografts were used in respectively, 5 and 5 studies covering 141 and 103 patients. Although not specified in all studies, across all three graft types most studies used a transphyseal surgical technique. To a lesser extent a partial epiphyseal/hybrid or all-epiphyseal technique was applied (Table 1).

### Risk of bias

The mean MINORS score for non-comparative studies and comparative studies were 9.1 ± 1.4 and 14.7 ± 3.6, respectively (Table 1). The full MINORS assessment is shown in appendix 2. The most common flaw was an unbiased (blinded) assessment of the study endpoints and all studies, except one, did not prospectively calculate a sample size.

### Primary outcome

**Fig. 2:**
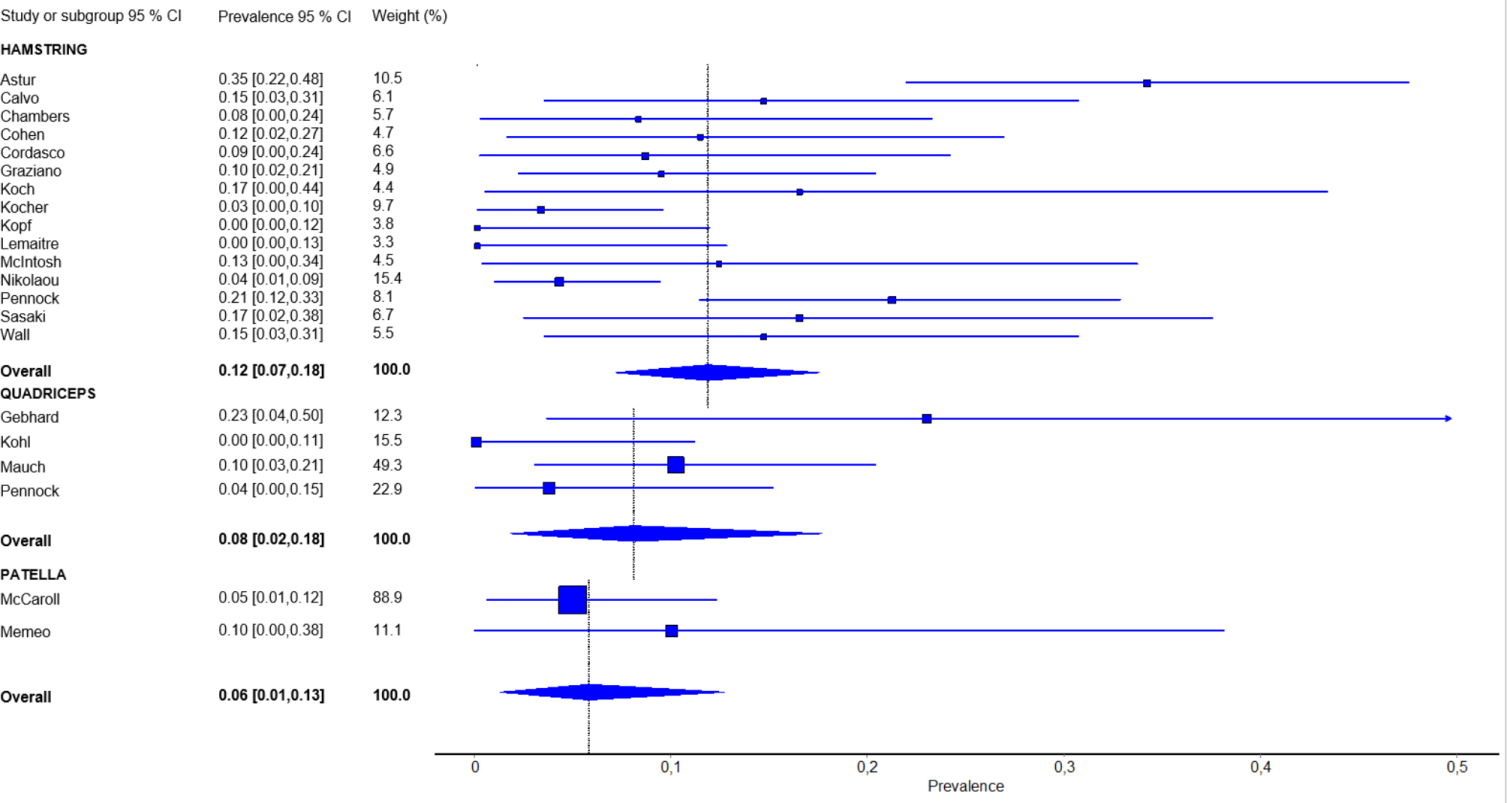
Pooled graft failure rates per graft type using the quality effects model. CI; confidence interval

In the meta-analysis, respectively 12 studies on ACLR with hamstring, 5 with quadriceps and 2 BPTB-grafts with comparable definitions of graft failure were included. The overall pooled failure rate was 12% for hamstring tendon autografts, 8% for quadriceps tendon autografts and 6% for BPTB autografts (Fig. 2). The overlapping 95% confidence intervals, suggest that the rates did not significantly differ between graft types. Sensitivity analyses indicated a relatively large contribution of the study by Astur. However, excluding this study would not change the interpretation of the pooled results (failure rate hamstring tendon autografts 10% [95% CI 6–14%].

Pennock et al. [48] was the only study that directly compared hamstring (n = 56) to quadriceps (n = 27) tendon autograft and found a statistically significant lower graft failure rate in favor of quadriceps tendon (p = 0.037).

Two studies [2, 44] were excluded from the analysis, mainly because they did not specify if the numbers provided were specifically regarding ipsilateral re-rupture or graft failure. The excluded studies reported similar failure rates though. The exact graft failure rates and used definitions are presented in Table 2.

**Table 2:** Graft failure rates, time to graft failure and definition of graft failure per graft type

Study	Graft failure rate (n, %)	Time to graft failure (months)	Definition of graft failure
Hamstring			
Astur [4]	18 (35)	mean 18.2, range 14–24	ACL graft re-rupture
Calvo [6]	4 (15.4)	7, 17, 35, 99	Traumatic rupture of ACL graft during contact sports
Chambers [7]	2 (8)	19.7, 49.5	ACL graft failure due to traumatic injury
Cohen [8]	3 (11.5)	4 (n = 1), > 12 (n = 2)	Traumatic rupture of ACL graft
Cordasco [10]	2 (8.7)	10, 18	Traumatic re-rupture
Graziano [29]	4 (9.5)	3, 10, 12, 24	Non-contact ACL injuries
Koch [33]	2 (16.7)	6–12 weeks, 24	Symptomatic graft failure
Kocher [34]	2 (3)	14, 21	Graft failure
Kopf [36]	0 (0)	.	Graft failure
Lemaitre [37]	0 (0)	.	Tear recurrence
McIntosh [41]	2 (12.5)	4, 24	Traumatic graft disruption
Nikolaou [45]	4 (4)	mean 16 (range 9–25)	Traumatic graft re-rupture
Pennock [48]	12 (21.4)	mean 18 ± 10.8	Graft failure
Sasaki [55]	3 (16.7)	mean 10.0 ± 1.6	Graft re-ruptures
Wall [66]	4 (14.8)	< 18	ACL graft failure
**Quadriceps**			
Gebhard [24]	3 (8)	.	Traumatic re-rupture ACL graft
Kohl [35]	0 (0)	.	Re-rupture
Mauch [39]	5 (10.2)	.	ACL Revision after adequate sports trauma
Pennock [48]	1 (3.7)	30	Graft failure
**BPTB**			
McCarroll [40]	3 (5)	.	Graft failure
Memeo [42]	1 (10)	.	ACL rupture after distortional trauma

ACL: anterior cruciate ligament, BPTB: bone-patellar-tendon-bone, n: number of patients, ±: standard deviation, if a ‘’.’’ is used, the study did not report this outcome

### Secondary outcomes

#### Knee stability

In the hamstring group, the KT-1000 was reported by 13 studies, ranging from 0.29 ± 1.07 mm to 2.58 (-2,7–7) mm side to side difference. In the BPTB group, two studies reported KT-1000 scores of 2.1 ± 1.2 mm and 3.3 ± 1.6 mm. McCarroll et al. (1994) reported a < 3.5 mm side to side difference in 51 patients, whereas 6 patients had a 4–5 mm difference (Table 3). In the quadriceps group, only Gebhard et al. (2006) reported a 2.0 mm difference (range 0–4 mm) (Table 3).

**Table 3:** Clinical examination per graft type

**Study**	**ROM**	KT-1000 side to side difference*, mean (± **SD or range)**	**Lachman (%)**	**Pivot shift (%)**
**Hamstring**				
Anderson [2]	n = 5 flexion deficit, mean 3°, none > 8°	1.5 mm ± 1.1 (0–3.75)	Negative (83), + 1 (17)	Negative (100)
Calvo [6]	.	2.6 mm (-2,7–7)	.	.
Cohen [8]	.	2.0 ± 1.0 mm	.	Negative (92), grade I (8)
Cordasco [10]	.	0.9 ± 0.5 mm	Negative (100)	Negative (100)
Faunø[22]	.	1.6 mm	.	.
Graziano [29]	.	1.1 ± 0.6 mm	Negative (98), positive (2)	Negative (98), positive (2)
Guzzanti [31]	.	1.2 mm (2–0)	.	.
Koch [33]	n = 4 reduced flexion of 5°–10°	1.5 ± 2.5 mm (-1–8)	.	.
Kocher [34]	.	.	Negative (86), + 1 (14)	Negative (95), grade I (5)
Kopf [36]	No ROM deficit	1.8 mm (1–3)	.	.
Lemaitre [37]	.	0.6 ± 1.4 mm	.	.
McIntosh [41]	.	.	Negative (94), + 1 (6)	.
Nikolaou [45]	No ROM deficit	2 mm (0–3.5)	+ 1 (97), + 2 (3)	Positive 2+ (2)
Razi [50]	.	.	Negative (61.1), positive (38.9)	Negative (100)
Redler [51]	.	0.3 ± 1.1 mm	Negative (100)	Negative (100)
Sasaki [55]	No ROM deficit	0.6 ± 0.9 mm	Negative (80), + 1 (20)	Negative (78), + 1 (22)
Seon [59]	No ROM deficit	.	.	.
Wall [66]	No ROM deficit	.	.	
**Quadriceps**				
Gebhard [24]	n = 2 loss in ROM (5%)	2.0 mm difference (0–4)	.	.
Kohl [35]	.	.	.	.
Shelbourne [61]	.	.		.
**BPTB**				
McCarroll [40]	.	< 3.5 mm difference (n = 51), 4–5 mm (n = 6)	.	.
Memeo [42]	No ROM deficit	3.3 ± 1.6 mm	Negative (100)	Negative (100)
Shelbourne [61]	No ROM deficit	2.1 ± 1.2 mm	Negative (100)	

* all studies except McCarroll[McCarroll] and Gebhard [24], which described the difference compared to the non-operated leg, describe the KT-1000 value of the operated leg, if a ‘’.’’ is used, the study did not report this outcome. BPTB: bone-patellar-tendon-bone, ROM: range of motion

Concerning stability tests, the Lachman and the Pivot shift at follow up were tests of interest. In the hamstring group, the Lachman test was reported by 9 studies and was negative in 80 to 100% of the cases. Five studies reported a positive Lachman ranging from 6 to 97% of the cases. The Pivot shift test was determined in 9 studies and was negative in 78–100% of the cases and positive in 2–22% of the cases. Two studies using quadriceps grafts reported a negative Lachman in 100% of the cases, one study also reported a negative Pivot shift in 100% of the cases (Table 3).

Of the 9 studies that described the range of motion (ROM) in the hamstring group, 7 studies reported no ROM deficit. Anderson et al. (2003) reported a flexion deficit with a mean of 3° in 5 patients (42%), but no greater than 8°. Koch et al. (2014) reported a slightly reduced flexion of 5°–10° in 4 patients (33%). In the quadriceps group, only Gebhard et al. (2006) reported a loss in ROM in 2 patients (5%) (Table 3).

#### Arthrofibrosis

Only 4 studies described the occurrence of arthrofibrosis. In the hamstring group, Kocher et al. (2007) reported 3 cases (5% of the study population) of arthrofibrosis 11 to 16 weeks postoperative. Nelson et al. (2016) reported 4 cases (1%) and Redler et al. (2012) confirmed there were no cases of arthrofibrosis. The definition of arthrofibrosis in all three studies was unclear. Additionally, in the BPTB group, McCarroll et al. (1994) reported 2 cases of arthrofibrosis (3%) and defined arthrofibrosis as > 5° extension loss compared with the non-operated knee.

#### Growth disturbances

In total 20 studies in the hamstring group, 4 in the quadriceps group and 2 in the BPTB group identified growth disturbances as an outcome measurement using radiographs. Two studies (Gicquel [26], Kopf [36]) were excluded from the analysis since only studies using radiographs were included.

In the hamstrings group, 8 studies reported no occurrence of length discrepancy, 13 studies no valgus, and 16 no varus or recurvatum deformity. Length discrepancy occurred in 10 studies ranging from a 1.2 to 22 mm difference compared to the other leg in 124 patients in total. Valgus deformity was reported by 6 studies, ranging from 0.46° to 4.5° in 64 patients in total. Varus deformity was reported by only 2 studies. Koch et al. (2014) reported one patient with a 1.5° varus deformity and Faunø et al. (2016) reported 23 patients with a varus deformity ≥ 2° on the operated knee, which was statistically significant compared to the non-operated knee (p = < 0.01). None of the studies reported the occurrence of a recurvatum deformity (Table 4).

**Table 4:** Growth disturbances per graft type

Study	Length discrepancy	Valgus deformity	Varus deformity	Recurvatum deformity
Hamstring				
Anderson [2]	2, 9, 10 mm (n = 3) -3 mm (n = 1)	0	0	0
Astur [4]	0	0	0	0
Calvo [6]	1–2 mm (n = 27)	0	0	0
Chambers [7]	3.0 mm (n = 3)	0.4 (MAD) (n = 3)	0	0
Cohen [8]	1–2 mm (n = 26)	0.46° (n = 26)	0	0
Cordasco [10]	5 mm (n = 6)	0	0	0
Faunø[22]	-3-4 mm (n = 33)	≥ 2° (n = 27)	≥ 2° (n = 23)	.
Graziano [29]	0	0	0	0
Guzzanti [31]	0	0	0	0
Koch [33]	5–10 mm (n = 4) >10 mm (n = 2)	4.5° (n = 1)	1.5° (n = 1)	.
Kocher [34]	0	0	0	0
Lemaitre [37]	.	> 4° (n = 2)	.	.
McIntosh [41]	6–7 mm (n = 15) 15 mm (n = 1)	0	0	0
Nikolaou [45]	0	0	0	0
Pennock [48]	0	0	0	0
Razi [50]	n = 1	n = 1	0	0
Redler [51]	0	0	0	0
Sasaki [55]	.	> 3° (n = 4)	.	.
Seon [59]	0	0	0	0
Willson [68]	12–22 mm (n = 2)	0	0	0
**Quadriceps**				
Gebhard [24]	0	.	.	.
Kohl [35]	-2-3 mm (n = 15)	n = 1	0	0
Mauch [39]	0	n = 1	0	0
Pennock [48]	0	0	0	0
**BPTB**				
Memeo [42]	0	0	0	0
Shelbourne [61]	0	0	0	0

* A value of 0 means that no growth disturbances were reported, ** Femoral transcondylar tangent, operated leg vs non-operated leg, *** Tibial transcondylar tangent, operated leg vs non-operated leg. MAD: Mechanical Axis Deviation, BPTB: bone-patellar-tendon-bone, if a ‘’.’’ is used, the study did not report this outcome

In the quadriceps group, Mauch et al. (2011) reported one patient with valgus deformity, Gebhard et al. (2006) reported no length discrepancies and Pennock et al. (2019) reported no length discrepancies as well as no angular deformities. Kohl et al. (2014) reported a mean length discrepancy of − 2.9 ± 8.6 mm (99.7 ± 0.9%) over all 15 patients compared to the other side with 2 patients > 10 mm and one patient with valgus deformity. Both Memeo et al. (2012) and Shelbourne et al. (2004) reported no growth disturbances in the BPTB group (Table 4).

#### PROMs

In the hamstring group, the (Pedi-)IKDC was reported by 16 studies, average scores ranged from 85.6 to 96.5. The Lysholm score was reported by 14 studies, ranging from 58.8 to 97.9. Nine studies used the Tegner score, showing results ranging from 4.9 to 8.6. Faunø et al. (2016) and Razi et al. (2019) reported a mean KOOS of 76.8 ± 15.1 and 75 ± 7.4, respectively. Cordasco et al. (2016) and Graziano et al. (2017) both reported the Marx Activity Rating Scale with mean values of 13.4 ± 3.6 and 23.2 ± 8.3, respectively (Table 5).

**Table 5:** PROMs per graft type, mean

**Study**	(pedi-*)IKDC	**Lysholm**	**Marx activity rating scale**	**KOOS**	**Tegner**
**Hamstring**					
Anderson [2]	96.5 ± 4.4 (range 86–100)	.	.	.	.
Astur [4]	.	Intact ACL vs. ACL graft failure (90.6 ± 6.1 vs. 58.8 ± 6.7) p = < 0.001	.	.	Intact ACL 7.0 ± 0.8, ACL graft failure 4.9 ± 1.3, p = 0.006
Calvo [6]	92 (range 44–100)	94 (range 55–100)	.	.	6 (range 3–9)
Chambers [7]	94.8 ± 5.3 (range 84.7–100)*	.	.	.	.
Cohen [8]	91.5 ± 5.7	93.5 ± 4	.	.	.
Cordasco [10]	94.6 ± 4.9	97.9 ± 4.0	13.4 ± 3.6	.	.
Faunø[22]	.	.	.	76.8 ± 15.1	6.1
Graziano [29]	93.1 ± 7.2	97.6 ± 4.5	23.2 ± 8.3	.	.
Koch [33]	88.5 (range 74.7–98.9)	93 (range 73–100)	.	.	6.2
Kocher [34]	89.5 ± 10.2	91.2 ± 10.7	.	.	.
Kopf [36]	95 (range 92–98)	96 (range 93–100)	.	.	.
Lemaitre [37]	83.3 ± 9.49	94.8 ± 6.39	.	.	.
McIntosh [41]	99 (range 94–100)	90 (range 74–94)	.	.	8.1 (range 7–9)
Nikolaou [45]	.	89 (range 77–100)	.	.	6 (range 4–8)
Pennock [48]	.	94 ± 6	.	.	7.1 ± 2.0
Razi [50]	85.6 ± 4.4	.	.	75 ± 7.4	.
Redler [51]	92.4 ± 10.0	94.4 ± 8.8	.	.	8.6 ± 1.4
Sasaki [55]	.	.	.	.	.
Seon [59]	.	97.8 (range 94–100)	.	.	.
Smoak [63]	94.89 ± 4.85 (range 90–100)	.	.	.	.
Wall [66]	94 ± 11 (range 49–100)	.	.	.	.
Willson [68]	96.0 ± 3.5 (range 89.1–100)*	.	.	.	.
**Quadriceps**					
Gebhard [24]	.	94.3 (range 53–100).	.	.	5.9
Kohl [35]	.	94.0 (range 68–100)			
Pennock [48]	.	96 ± 8	.	.	6.6 ± 1.6
**BPTB**					
Shelbourne [61]	95.4 ± 6.9	.	.	.	

* used pedi-IKDC scale. IKDC: international knee documentation committee, KOOS: knee injury and osteoarthritis outcome score, ACL: anterior cruciate ligament, BPTB: bone-patellar-tendon-bone, ±: standard deviation, (): range, if a ‘’.’’ is used, the study did not report this outcome

In the quadriceps group, the Lysholm score was reported by three studies. Gebhard et al. (2006), Kohl et al. (2014) and Pennock et al. (2019) reported a mean Lysholm score of 94.3 (range 53–100), 94.0 (range 68–100) and 96 ± 8, respectively. Gebhard et al. (2006) and Pennock et al. (2019) also reported a mean Tegner score of 5.9 and 6.6, respectively (Table 5).

Pennock et al. (2019) also compared the Lysholm and Tegner scores of the quadriceps group directly to the hamstring group, showing no statistically significant differences (p = 0.095, p = 0.229, respectively).

In the BPTB group, only Shelbourne et al. (2004) reported a mean IKDC score of 95.4 ± 6.9 (Table 5).

### Return to sports

In total 19 studies described return to sports (RTS) as an outcome measurement. Most studies recorded the percentage of patients who returned to the same level or higher level of sports or activity as before the operation ranged. These percentages ranged from 63 to 100% in the hamstrings group (n = 15) and 92–100% in the BPTB group. No studies in the quadriceps group identified RTS as an outcome measurement. Five studies in the hamstring group reported the time to RTS, with means ranging from 7 to 13.5 months. In the BPTB group, only McCarroll et al. (1994) reported a time to RTS ranging from 1 to 9 seasons of competition (Table 6).

**Table 6:** Return to sports per graft type

**Study**	**Percentage**	**Time to return to sports [months ± SD (range)]**	**Definition**
**Hamstring**			
Anderson [2]	92%	.	Perform very strenuous activities (IKDC)
Astur [4]	93%	Intact ACL 7.4 ± 1.1 (6–9), ACL graft failure 7.5 ± 1.2 (6–9)	Return to activity
Calvo [6]	89%	.	Return to the patients’ previous sports activity
Chambers [7]	100%	11.3 ± 3.3 (9–23)	Return to unrestricted athletics
Cohen [8]	89%	.	Return to the same level of sports activity as before the injury
Cordasco [10]	96%	13.5 (8–22)	Return to unrestricted competitive sports after successful completion of the QMA and RTS performance analysis
Graziano [29]	93%	12 ± 2.0	Athletes were cleared for return to sports based on quantitative measures using the limb symmetry index and qualitative measures (QMA) as well as the ability to meet the demands of their sport.
Guzzanti [31]	100%	.	Return to high-level sports activity equiv- alent to the patients’ preinjury status
Kocher [34]	100%	.	Return to cutting or pivoting sports
McIntosh [41]	63%	.	Return to the patients identical preoperative sport
Nikolaou [45]	78%	.	Return to the patients’ identical preoperative sport
Redler [51]	100%	.	Return to full activity, including sports that involve cutting
Seon [59]	91%	.	Return to preinjury sports activity levels
Wall [66]	81%	.	Return to sports and recreational activities
Willson [68]	83%	8 (6–14)	Return-to-play assessment including clinical parameters, subjective outcomes (Pedi-IKDC, ACL-RSI), symmetric quadriceps and hamstring strength (< 10% deficit on iso- metric and isokinetic strength testing), and multiple hop testing (as evaluated by a sports-trained physical therapist with an assessment of distance, symmetry, and form)
**BPTB**			
McCarroll [40]	92%	1–9 seasons of competition after surgery	Return to the patients’ previous sports at the same level of competition
Memeo [42]	100%	.	Return to the patients’ preinjury level of daily activity and athletic participation
Shelbourne [61]	100%	.	Return to competitive sports

SD: standard deviation, ACL: anterior cruciate ligament, (pedi)-IKDC: (pediatric) international knee documentation committee, QMA: quality of movement assessment, RTS: return to sports, ACL-RSI: Anterior Cruciate Ligament Return to Sport after Injury (ACL-RSI) score, BPTB: bone-patellar-tendon-bone, if a ‘’.’’ is used, the study did not report this outcome

## Discussion

The most important finding of this systematic review was that no specific autograft type seems superior regarding graft failure rates in skeletally immature children after transphyseal, all-epiphyseal or partial epiphyseal ACL reconstruction. Due to the lack of studies evaluating quadriceps or BPTB autografts, quantitative comparison of other outcomes than graft failure was not possible. Hamstring tendon autograft was evaluated most frequently. Graft failure rates of hamstring tendon autografts were 12% (95%-CI: 7-18%), quadriceps tendon 8% (95%-CI: 2-18%) and BPTB 6% (95%-CI: 1-13%). There were no statistically significant differences in graft failure rates between the three graft types based on comparison of the 95%-confidence intervals. The variability in time to graft failure was high. According to the available literature in this systematic review, no graft seems therefore to be evidently superior in graft failure rates in transphyseal, all-epiphyseal or hybrid ACL reconstruction techniques. Overall, there are no large differences in graft failure rates, however there is a tendency towards higher failure rates in hamstring tendon autografts. This tendency is supported by the only study in this review that directly compared hamstring autografts to quadriceps autografts, showing a statistically significant lower graft failure rate when using a quadriceps tendon autograft [48]. A systematic review on graft types in the population under 19 years of age that included both skeletally immature and mature patients also supports these findings [11].

Compared to previous systematic reviews on outcomes after ACLR in skeletally immatures, graft failure rates were somewhat higher in the current study [23, 38, 70]. Compared to systematic reviews including patients under 19 years of age, the graft failure rates were lower in the current review [11, 32]. Although these systematic reviews show no statistically significant difference between the techniques in terms of graft failure or growth disturbances, they did use different in- and exclusion criteria and included multiple graft types, making it difficult to compare the results directly [17, 23, 38, 70].

One of the secondary outcomes of interest were growth disturbances. After ACL reconstruction, growth disturbances can occur in about 2.6–24% of the cases [22, 38]. In contrast to the systematic review by Collins et al. [9], the current systematic review found that length discrepancies, although in many cases very limited, were more common than valgus angular deformities [9]. In the systematic review by Frosch et al. [23], hamstring autografts were associated with a slightly lower risk of the occurrence of leg-length differences or axis deviations compared to BPTB grafts [23]. Regarding recurvatum deformities, the International Olympic Committee (IOC) statement discourages the use of BPTB grafts due to damage to the tibial tubercle apophysis when harvesting the graft. Frosch et al. [23] did not state this, but hypothesized that the apophysis of the tibia could be damaged due to the position of the tibial drill tunnel in physeal-sparing techniques or that the transplant blocks the tibial growth plate when it’s fixated on the ventral side [3, 23]. No recurvatum deformities were reported in the included studies that used BPTB in this systematic review. In both studies the follow-up did however not include a long leg radiograph, which is a limitation in diagnosing growth disturbances after ACLR [42, 61].

Another secondary outcome of interest was return to sports. The RTS percentages were high in most studies, with only two studies scoring lower than 80% [41, 45]. Interestingly both these studies reported a discrepancy between RTS percentages and return to previous levels of activity according to the Tegner score, which was above 80% in both studies [41, 45]. The lack of a clear and comparable definition of RTS in the different studies makes interpretation challenging. Overall, it seems that RTS percentages in skeletally immature patients after ACLR are high and independent of graft type [25, 30]. However, not all patients return to the exact same type of sports, but most patients are able to return to a similar level of sport [13, 32].

The other secondary outcomes of interest could also not be quantitatively compared between different graft types, due to limited number of studies evaluating a specific outcome or due to the use of different outcome measures. In all graft groups, clinical evaluation showed limited residual loss of range of motion and instability in the KT-1000 arthrometer or during Lachman or Pivot shift test. Different knee-specific and physical activity PROMs were analyzed in the included studies, which showed similar outcomes in the graft groups. Mostly adult versions of PROMS were used, while these are often not appropriate for a pediatric population [15].

A limitation of this systematic review is that it focused only on clinical outcomes of graft types in ACL reconstruction techniques in skeletally immature patients. Other outcomes, such as incorporation, ligamentalization and mechanical properties of the different graft types were not included. These processes are relevant in relation to the structural properties of a graft [1]. As such they might be important to determine which graft type is best suited to act as an ACL surrogate in terms of stability and mechanical durability. A stronger graft may be able to resists loads that caused the native ACL to fail. However, if a surrogate tissue is too stiff, it could potentially lead to joint overstraint and increased risk of osteoarthritis or early graft failure. Additionally, the mechanical properties of the ACLR grafts have been demonstrated to decrease during the postoperative ligamentalization process and never recover. A stronger graft may remain sufficient as surrogate after this process, while a graft similar to a native ACL may remain insufficient [14]. There have been studies in the past investigating these processes. Aitchinson et al. [1] showed that quadriceps autografts have an improved graft maturation, remodeling and structural integrity compared to hamstring autografts at one year postoperatively [1]. The clinical relevance remains unclear since more graft failures seem to occur after 12 months postoperatively [4, 16, 44]. Schmidt et al. [58] conducted a cadaveric cohort study on mechanical properties and microstructures of ACLs and grafts in skeletally immature specimens. The study shows that patellar tendons were most similar to native ACLs mechanical properties, which corresponds to the findings in adults in a cadaveric study by Schilaty et al. [14, 56]. Semitendinosus and iliotibial bands (ITBs) were significantly stronger but less compliant than quadriceps or patellar tendons [14]. ITBs were the most similar to ACLs regarding microstructure [14].

Another limitation is the methodological quality of the included studies. Since almost all studies are cohort studies or case series, the level of evidence is low. The MINORS tool used to determine the quality of the studies also showed moderate quality. The most common factors which increased the risk of bias were an unbiased (blinded) assessment of the study endpoints and failing to prospectively calculate a sample size. The different graft types could not be compared directly due to variations in outcomes and surgical techniques and due to the small sample sizes of the quadriceps and BPTB groups. The strength of this systematic review is however that it provides a current overview of the outcomes of different graft types used in skeletally immature patients. The investigated groups in the individual studies were grossly comparable regarding surgical indications, study designs and follow-up duration.

Future research should focus on the clinical outcomes and complications of using a quadriceps or BPTB autograft directly compared to a hamstring autograft. Another field of interest might be extra-articular surgical techniques such as the ‘’over-the-top ACL reconstruction or additional lateral extra-articular tenodesis (LET). ACLR in immature patients following these techniques also showed promising functional results in terms of return to sports and graft survival rate [e.g., 51]. The use of a registry such as the ESSKA Paediatric Anterior Cruciate Ligament Initiative (PAMI) registry might provide an opportunity to investigate a large number of patients [43]. Additionally, future research should also focus on graft characteristics including ligamentalization and mechanical properties, this might be insightful when choosing a specific graft type in skeletally immature children.

## Conclusions

Based on this review it is not possible to determine a superior graft type for ACLR in skeletally immature. Of the included studies, the most common graft type used was the hamstring tendon. Overall, graft failure rates are low, and most studies show good clinical outcomes with high return to sports rates.

### Electronic supplementary material

Below is the link to the electronic supplementary material.

**Appendix 1**: Draft Pubmed search. **Appendix 2**. Study quality and study bias scoring of the 30 included studies using the MINORS score for nonrandomized studies

## Data Availability

All data generated or analyzed during this study are included in this published article [and its supplementary information files].

## Abbreviations

ACL: Anterior Cruciate Ligament
ACLR: Anterior Cruciate Ligament Reconstruction
ACL-RSI: Anterior Cruciate Ligament Return to Sport after Injury score
BMI: Body Mass Index
BPTB: Bone-patellar-tendon-bone
IOC: International Olympic Committee
ITB: Iliotibial band
KOOS: Knee injury and Osteoarthritis Outcome Score
MAD: Mechanical Axis Deviation
MeSH: Medical subject headings
MINORS: Methodological Index for Non-Randomized Studies
MRI: Magnetic Resonance Imaging
PAMI: Paediatric Anterior Cruciate Ligament Initiative
(pedi-)IKDC: (Pediatric) International Knee Documentation Committee
PRISMA: Preferred Reporting Items for Systematic Reviews and Meta-Analyses
PROMs: Patient reported outcome measures
PROSPERO: Prospective register of systematic reviews
ROM: Range of motion
RTS: Return to sports
SD: Standard deviation
QMA: Quality of movement assessment
