# Supplementary material for: Clinical outcomes of different autografts used for all-epiphyseal, partial epiphyseal or transphyseal anterior cruciate ligament reconstruction in skeletally immature patients – a systematic review
Source: BMC Musculoskelet Disord. 2023 Aug 3;24:630. doi: 10.1186/s12891-023-06749-4 (PMC10401849; doi:10.1186/s12891-023-06749-4)
Supplement: Supplementary file 1 — Appendix 1: Draft Pubmed search. Appendix 2. Study quality and study bias scoring of the 30 included studies using the MINORS score for nonrandomized studies [file 12891_2023_6749_MOESM1_ESM.pdf]

Appendix

Appendix 1: Draft Pubmed search

Search	Query	Results
**#4**	Search: **#1 AND #2 AND #3** Sort by: **Publication Date**	153
**#3**	Search: **Anterior Cruciate Ligament[Mesh] OR Anterior Cruciate Ligament Reconstruction[Mesh] OR Anterior Cruciate Ligament Injuries[Mesh] OR "anterior cruciate ligament*"[tiab] OR "cranial cruciate ligament*"[tiab] OR "ACL reconstruction"[tiab] OR "ACL repair*"[tiab] OR "ACL surger*"[tiab] OR transphyseal[tiab] OR "all-epiphyseal"[tiab] OR "partial epiphyseal"[tiab] OR "physeal sparing"[tiab]** Sort by: **Publication Date**	25,306
**#2**	Search: **"Bone-Patellar Tendon-Bone Grafts"[Mesh] OR "Bone-Patellar Tendon-Bone Grafting"[Mesh] OR "Hamstring Tendons"[Mesh] OR "Quadriceps Muscle"[Mesh] OR "Autografts"[Mesh] OR "Patellar Ligament"[Mesh] OR "bone-patellar-tendon-bone*"[tiab] OR "bone patellar*"[tiab] OR "soft tissue autograft*"[tiab] OR "hamstring tendon*"[tiab] OR "quadriceps muscle*"[tiab] OR autograft*[tiab] OR "patellar ligament*"[tiab] OR "patellar tendon*"[tiab] OR "patella tendon*"[tiab] OR "ligamentum patellae"[tiab]** Sort by: **Publication Date**	36,684
**#1**	Search: **"Growth Plate"[Mesh] OR "growth plate*"[tiab] OR "open phys*"[tiab] OR "epiphyseal plate*"[tiab] OR "skeletally immature"[tiab] OR "open growth plate*"[tiab] OR autotransplant*[tiab] OR "autogenous graft*"[tiab]** Sort by: **Publication Date**	20,315

Appendix 2. Study quality and study bias scoring of the 30 included studies using the MINORS score for nonrandomized studies

Study	A clearly stated aim	Inclusion of consecutive patients	Prospective collection of data	Endpoint appropriate for aim of study	Unbiased assessment of study endpoint	Follow-up period appropriate	Loss of follow-up less than 5%	Prospective calculation of study size	Adequate control group	Contemporary group	Baseline equivalence of groups	Adequate statistical analysis
Anderson[2]	1	1	0	2	0	2	2	0				
Astur[4]	2	1	2	2	1	2	1	0	0	2	2	2
Calvo[6]	2	2	0	2	0	2	2	0				
Chambers[7]	2	2	0	2	0	2	2	0				
Cohen[8]	2	1	0	1	0	2	2	0				
Cordasco[10]	2	2	2	2	0	2	2	0				
Faunø[21]	2	1	0	2	0	2	1	0				
Gebhard[23]	1	1	0	2	0	2	2	0	0	1	0	1
Gicquel[25]	2	1	0	2	0	2	0	0	1	2	0	2
Graziano[28]	2	1	0	2	0	0	1	0				
Guzzanti[30]	2	1	0	2	0	2	2	0				
Koch[32]	2	1	2	2	0	2	2	0				
Kocher[33]	2	2	0	2	0	2	1	0				
Kohl[34]	2	1	0	2	0	2	2	0				
Kopf[35]	2	1	0	2	0	2	2	0				
Lemaitre[36]	2	1	0	2	0	2	1	0				
Mauch[38]	2	2	0	2	0	2	2	0				
McCarroll[39]	2	2	0	2	0	2	2	0				
McIntosh[40]	2	2	0	2	0	2	2	0				
Memeo[41]	0	1	0	1	0	2	2	0				
Nelson[43]	2	1	2	2	0	2	1	0				
Nikolaou[44]	2	2	0	2	0	2	2	0				
Pennock[47]	2	1	0	2	0	2	1	0	2	2	2	2
Razi[49]	1	2	0	2	2	2	1	0	0	2	2	2
Redler[50]	2	2	0	2	0	2	1	0				
Sasaki[55]	2	2	2	2	0	2	2	2	2	2	0	2
Seon[59]	1	1	0	2	0	2	2	0				
Shelbourne[61]	2	1	2	2	0	2	2	0				
Smoak[63]	2	1	0	2	0	2	1	0				
Wall[66]	2	2	0	2	0	2	1	0				
Willson[68]	2	2	0	2	0	2	2	0				
